# Sustained remission for mycosis fungoides following duvelisib and mogamulizumab

**DOI:** 10.1016/j.jdcr.2026.06.008

**Published:** 2026-06-15

**Authors:** Kiera Murphy, Christopher Bazewicz

**Affiliations:** a College of Medicine, Penn State University, Hershey, Pennsylvania; b Department of Dermatology, Penn State University, Hershey, Pennsylvania

**Keywords:** duvelisib, mogamulizumab, mycosis fungoides

## Introduction

Mycosis fungoides (MF) is the most common cutaneous T-cell lymphoma, often requiring combinatorial therapy in advanced-stage disease. Mogamulizumab, a C-C chemokine receptor 4 (CCR4) monoclonal antibody, and duvelisib, a phosphoinositide 3-kinase (PI3K) δ/γ inhibitor, each demonstrate efficacy through antiproliferative effects as well as immune modulation but can trigger immune-related adverse events (iRAEs).^1^, ^2^, ^3^ Their concurrent use for MF has not, to our knowledge, been reported. Herein, we describe their use for tumor-stage MF with CD30^−^ large-cell transformation (LCT).

## Case report

**Fig 1:**
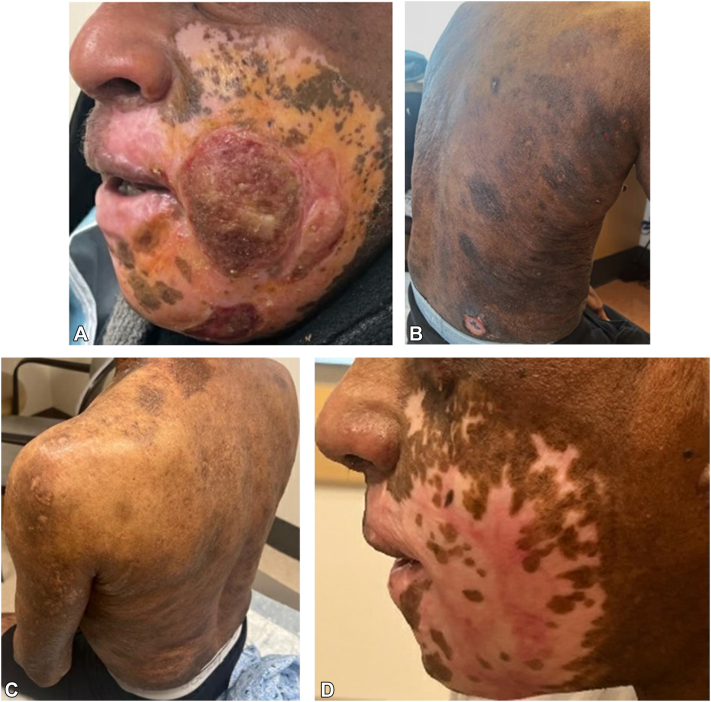
Clinical response in advanced-stage MF with CD30^−^ LCT. **A,** Large ulcerated tumor with dyspigmentation involving the left cheek at initial presentation. **B,** Widespread cutaneous disease (BSA = 44%; mSWAT = 48) prior to initiation of duvelisib and mogamulizumab combinatorial therapy. This representative image shows scattered violaceous to hyperpigmented patches and an ulcerated tumor on the back. **C, D,** After 2 months of combinatorial therapy, the patient had marked reduction in overall disease burden (BSA = 3%; mSWAT = 6). These representative images show scarring and post-inflammatory hyperpigmentation in areas previously occupied by MF.

A 67-year-old male with advanced-stage MF presented to the clinic for evaluation. The patient had 40% to 50% body surface area (BSA) involved by patches, plaques, and several tumors, including a large, ulcerated tumor involving his left cheek (Fig 1, *A*), and dermatopathic lymph nodes on excisional lymph node biopsy (T3N1M0B0 disease; stage IIB). The tumor of the left cheek revealed CD30^−^ (<1% CD30 staining) LCT. The patient was not responding to brentuximab vedotin at this time. Previous treatments included topical steroids, narrow-band UVB phototherapy, targeted and total skin electron beam radiation, 0.04% carmustine ointment, 5% imiquimod cream, 0.016% mechlorethamine gel, and oral bexarotene.

At this appointment, the decision was made to stop brentuximab, continue topical steroids, and initiate the patient on romidepsin and targeted radiation for the tumor of the left cheek. Targeted radiation was successful (8 gray; 2 fractions), but the patient was unable to tolerate romidepsin, even at a reduced dose. The patient was subsequently initiated on 15 mg twice a day duvelisib and standard dosing of mogamulizumab 1 month following cessation of romidepsin therapy (Table I).

**Table I:** Treatment timeline and clinical course

Date	Event
August 20, 2023-October 20, 2023	Duvelisib 15 mg twice a day + standard dosing mogamulizumab initiated; discontinued for transaminitis (ALT/AST 411/550)
October 20, 2023-November 17, 2023	All therapy held; 1-month prednisone taper initiated with resolution of transaminitis
November 17, 2023-November 20, 2023	Duvelisib 15 mg daily initiated; discontinued for transaminitis (ALT/AST 47/88)
November 20, 2023-December 1, 2023	Treatment held with resolution of transaminitis
December 1, 2023-December 23, 2023	Duvelisib 15 mg every other day initiated; discontinued for transaminitis (ALT/AST 93/158) which subsequently resolved
January 26, 2024-April 19, 2024	Mogamulizumab initiated at standard dosing; discontinued for severe transaminitis (ALT/AST >1000)
April 19, 2024-May 17, 2024	Treatment held; 1-month prednisone taper initiated with resolution of transaminitis
May 17, 2024-January 23, 2026	Sustained remission off all therapy until confirmed tumoral relapse on January 23, 2026

*ALT*, Alanine aminotransferase; *AST*, aspartate aminotransferase.

After 2 months of combinatorial therapy, the patient had a dramatic improvement in skin disease burden (Fig 1, *B-D*). Specifically, his %BSA and modified Severity Weighted Assessment Tool (mSWAT) score went from 44% and 48 to 3% and 6, respectively. However, the patient developed a transaminitis (Alanine aminotransferase = 411 units/L; Aspartate aminotransferase = 550 units/L), which responded to a 1-month prednisone taper and cessation of therapy. The patient was then trialed on duvelisib monotherapy (15 mg/day then every other day), but this was discontinued due to the development of a moderate transaminitis. Due to tumoral relapse in the skin, the patient was trialed on monotherapy with mogamulizumab. Following 3 months of therapy, the patient developed a significant transaminitis (Alanine aminotransferase/Aspartate aminotransferase >1000 units/L), with whole-body positron emission tomography/computed tomography showing evidence of gastritis and pneumonitis.

Consultations with pulmonology, hepatology, and gastroenterology were placed, and consensus regarding the autoimmune nature of these findings was made based on the known side effect profile of mogamulizumab and duvelisib, the fact that the patient’s previous transaminitis resolved quickly following the initiation of prednisone, elevation in serum IgG (1746 mg/dL) testing with a negative viral hepatitis workup, and confirmation of gastritis via biopsy. Thus, another 1-month prednisone taper was initiated, and mogamulizumab was stopped. Following this prednisone taper, the patient maintained a complete remission for roughly 20 months off all therapy with resolution of laboratory abnormalities prior to relapse.

## Discussion

Mogamulizumab is a CCR4 monoclonal antibody that exerts its effect by both targeting neoplastic lymphocytes for antibody-dependent cellular cytotoxicity as well as depleting regulatory T cells.^1^ It is most effective for MF patients with greater degrees of blood involvement and those with Sezary syndrome (SS), but it can be used in patients with skin-predominant disease, as in this case.^2^ Duvelisib is a PI3K δ/γ inhibitor that has shown efficacy for MF/SS.^3^^,^^4^ This is through both inhibition of the PI3K/AKT/mTOR pathway, which is overactive in a subset of patients with MF/SS, as well as through regulatory T-cell depletion and inhibition of M2 macrophage polarization.^5^^,^^6^ Both mogamulizumab and duvelisib can lead to iRAEs, an effect likely potentiated with their combination, as in this case.^7^ This immune activation, in part through regulatory T-cell depletion and inhibition of M2 macrophage polarization, likely also led to the sustained remission seen off all therapy.

This case highlights the potential utility of mogamulizumab and duvelisib for relapsed/refractory MF/SS. To the authors’ knowledge, this is the first report in the literature detailing their combination for MF. The patient’s sustained remission off all therapy is also noteworthy in the setting of tumor-stage disease with CD30^−^ LCT, which is associated with a very poor prognosis.^8^ Due to the potential for enhanced iRAEs with this combination, frequent laboratory monitoring with a focus on liver function testing, as well as a comprehensive review of systems to detect other iRAEs, is key to its successful administration. While this regimen induced several iRAEs, these were manageable with therapy cessation and systemic steroids.

## Conflicts of interest

None disclosed.
